# Nonabsorbable suture granuloma mimicking ovarian cancer recurrence at combined positron emission tomography/computed tomography evaluation: a case report

**DOI:** 10.1186/1752-1947-8-202

**Published:** 2014-06-18

**Authors:** Ludovica Imperiale, Claudia Marchetti, Laura Salerno, Roberta Iadarola, Carlotta Bracchi, Laura Vertechy, Lucia di Francesco, Angela Musella, Elisa Bevilacqua, Primo Pennesi, Innocenza Palaia, Pierluigi Benedetti Panici

**Affiliations:** 1 Department of Gynecological and Obstetrical Sciences and Urological Sciences, Sapienza University of Rome, Via del Policlinico, 155, 00161 Rome, Italy

**Keywords:** Cancer, Diagnosis, Foreign body, Surgery

## Abstract

**Introduction:**

This is the first case of suture granuloma mimicking isolated ovarian cancer relapse. Only six analogous cases have been previously reported in other malignancies.

**Case presentation:**

We report the case of a 44-year-old Caucasian woman with partially platinum-sensitive ovarian cancer in which radiological features, including computed tomography and combined ^18^F-fluorodeoxyglucose-positron emission tomography/computed tomography, were strongly suggestive of isolated cancer relapse in her right subdiaphragmatic region. Laparoscopic examination resulted negative, but was not completely suitable due to widespread adhesive syndrome. The laparotomy for secondary cytoreductive surgery and biopsy of the suspected area showed inflammatory granuloma caused by nonabsorbable propylene suture, without evidence of neoplastic cells. Moreover, unexpected peritoneal carcinosis was found.

**Conclusions:**

This evidence suggests that clinical details about previous surgical procedures are necessary for adequate interpretation. Although much progress has been made in imaging techniques, especially in the promising field of combined ^18^F-fluorodeoxyglucose positron emission tomography/computed tomography, these procedures should be still thoroughly investigated in order to promptly rule out tumor recurrence and avoid unnecessary surgery.

## Introduction

Despite aggressive and curative initial treatment, the majority of patients with ovarian cancer manifest persistent disease or develop fatal recurrence. The liver is the fourth most common site of metastasis in recurrent ovarian cancer (ROC) [1]: clinical data from living patients have shown an incidence of hepatic metastasis of approximately 9.4% [2]. Currently available therapeutic options for recurrent patients are second-line chemotherapy and secondary cytoreductive surgery (SCR); these options are reserved to selected patients in whom the number of metastatic sites is limited and complete surgical resection is an expected outcome [3,4].

The occurrence of granuloma after surgery with nonabsorbable surgical suture has been rarely described after bowel, lung and pharynx oncologic surgeries [5-7]; in all these cases granuloma was confused with cancer recurrence and patients received unnecessary surgeries.

To the best of our knowledge this is the first case of nonabsorbable suture granuloma mimicking ovarian cancer recurrence after primary debulking surgery for ovarian cancer.

We report the case of a woman with partially platinum-sensitive ovarian cancer in which radiological features, including computed tomography (CT) and combined ^18^F-fluorodeoxyglucose (^18^F-FDG) positron emission tomography/CT (PET/CT), were strongly suggestive of isolated cancer relapse in her right subdiaphragmatic region.

## Case presentation

A 44-year-old Caucasian woman was admitted to our institution because of adnexal complex masses, ascites and a carbohydrate antigen (CA)-125 value of 876U/mL (normal range 0 to 35U/mL).

Apart from a previous caesarean section, her gynecologic history was uneventful. She underwent laparoscopic evaluation before attempting cytoreductive surgery; she was considered not eligible for primary debulking surgery because of mesenterial involvement and diffuse peritoneal carcinosis with extension to her upper abdomen. Biopsies documented a moderately differentiated papillary ovarian carcinoma at histological findings; according to FIGO (International Federation of Gynecology and Obstetrics) staging it corresponded to IIIC final stage. After three courses of neoadjuvant chemotherapy based on a standard tri-weekly carboplatin and paclitaxel schedule, she achieved a partial clinical response and underwent interval debulking surgery with residual tumor smaller than 0.5cm. Consequently, she completed the chemotherapy administration receiving three more cycles of adjuvant treatment (carboplatin and paclitaxel).

**Figure 1:**
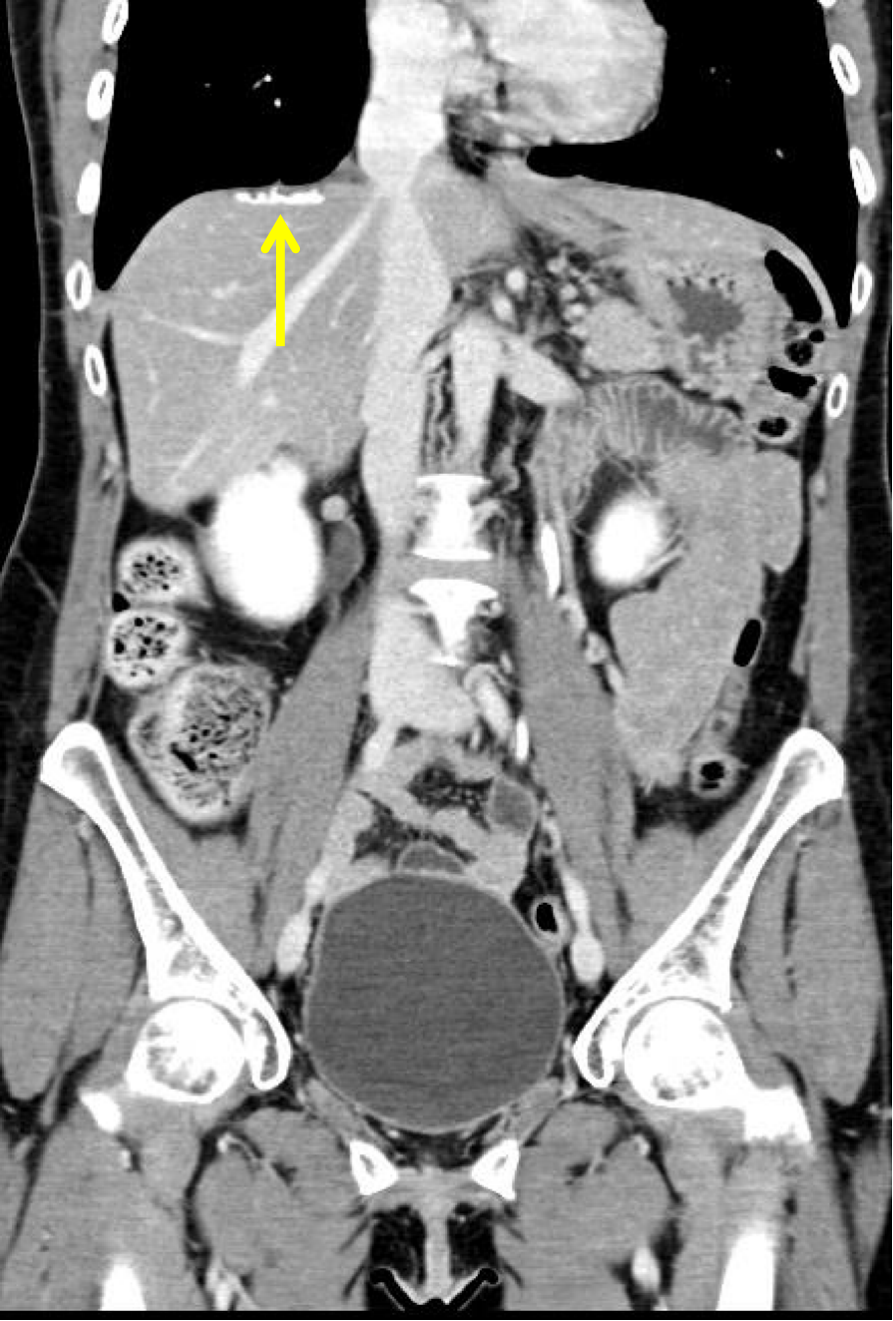
Computed tomography scan of hyperdense lesion in the right subdiaphragmatic region and a plaque thickening of the diaphragmatic dome (arrow).

**Figure 2:**
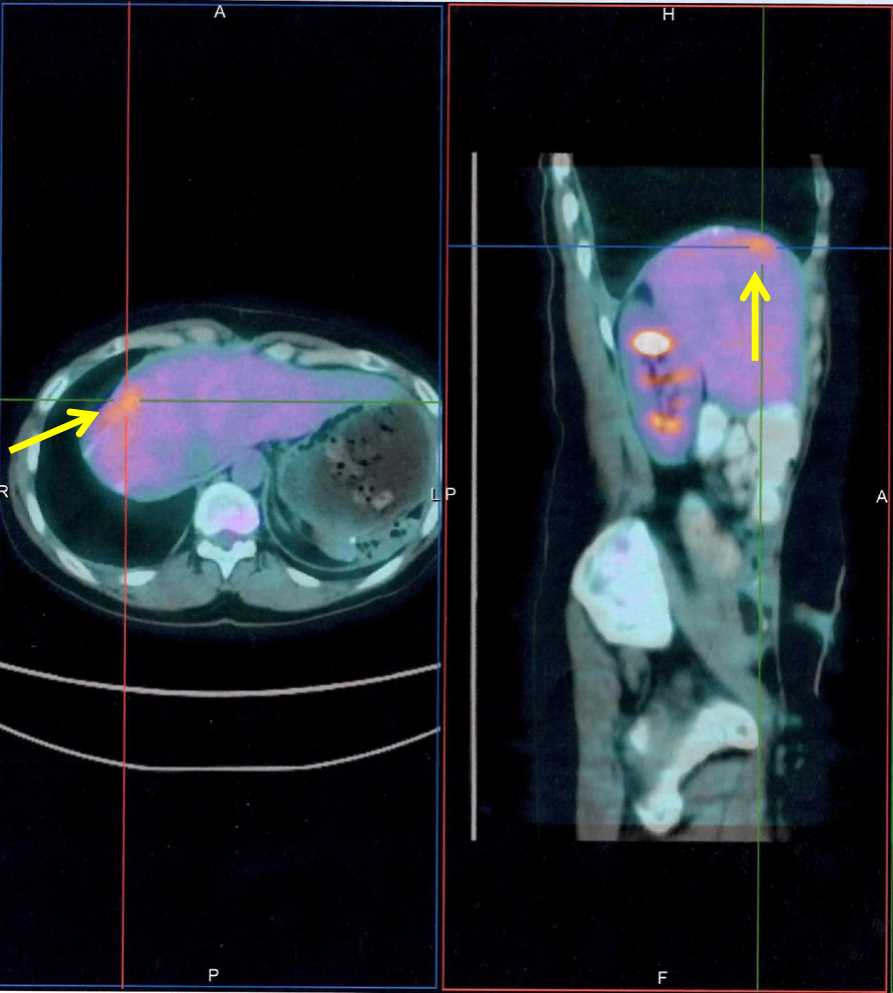
Positron emission tomography/computed tomography scan showing isolated high metabolic uptake (SUV: 4.2) in the right subdiaphragmatic region (arrows).

**Figure 3:**
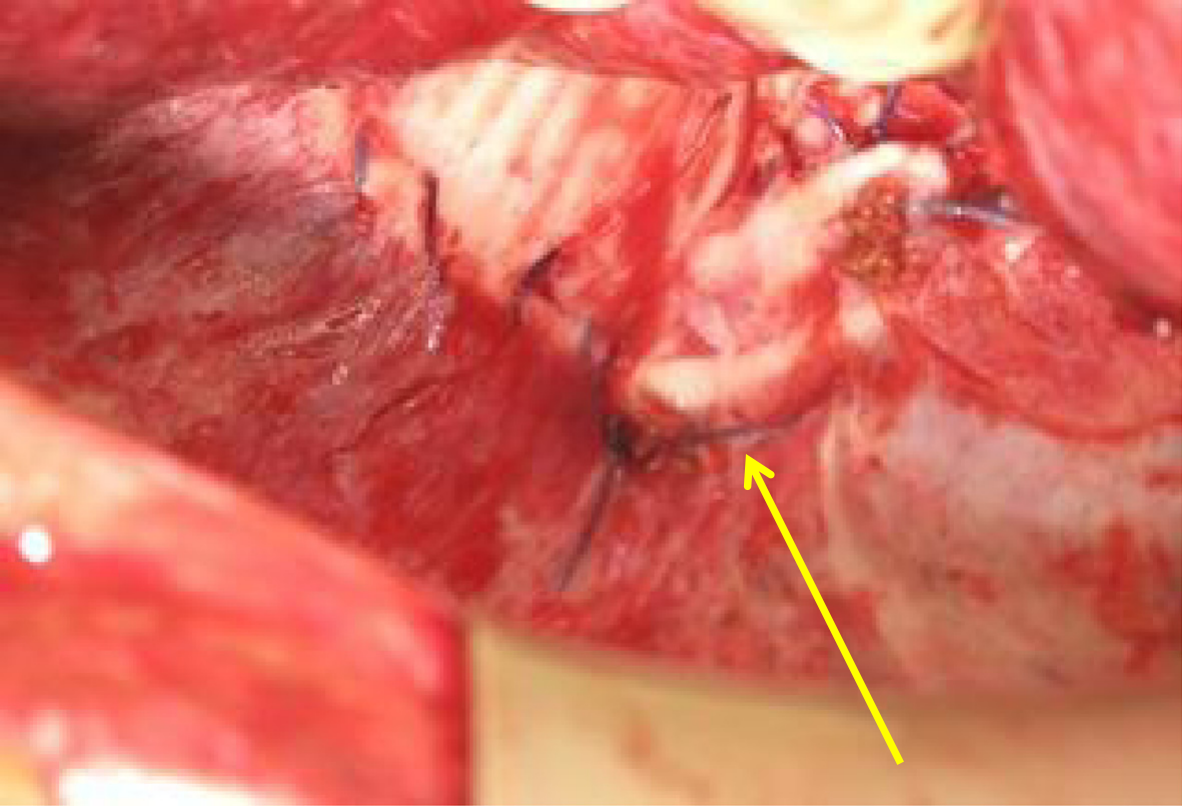
Granuloma from propylene stitch suture (arrow) applied at the time of the interval debulking surgery.

Further follow-ups were negative for the next 8 months, when a rise of CA-125 to 201U/mL was documented. Laboratory data from liver functions tests were unremarkable; no predisposing factors for hepatic cirrhosis, steatohepatitis or dysmetabolic diseases were identified.She was submitted to an abdominopelvic CT scan (Figure 1) which showed a hyperdense lesion in her right subdiaphragmatic region and a plaque thickening of her diaphragmatic dome, without evidence of peritoneal carcinosis. Taking into account her clinical condition and the isolated area of recurrence considered, she was judged eligible for SCR. In order to achieve a more accurate presurgical assessment a whole-body PET/CT (Figure 2) was performed, showing high metabolic uptake with standardized uptake value (SUV) up to 4.2 in the suspected area, without evidence of other sites of disease.She underwent surgery. The laparoscopic examination resulted negative, but was not completely reliable due to severe adhesive syndrome. A laparotomy biopsy of the area situated in her right subdiaphragmatic region, over her hepatic dome was performed. A frozen section revealed an inflammatory granuloma probably derived from a propylene stitch applied at the time of the interval debulking surgery (Figure 3). Furthermore an unexpected diffuse peritoneal carcinosis, extended to her entire abdominal cavity but without involvement of her upper abdomen, was identified and multiple biopsies were carried out.

Definitive histological findings confirmed that the subdiaphragmatic lesion was an inflammatory granuloma, characterized by epithelioid histiocytes and giant cells, with lymphocyte infiltration, around a nonabsorbable propylene suture derived from previous surgery, while other biopsies were found to be sites of relapse.

After surgery she was submitted to second-line chemotherapy based on carboplatin and Caelyx^®^ (doxorubicin hydrochloride) and she is now under third-line chemotherapeutic treatment still with persistent evidence of disease.

## Discussion

The role of cytoreduction in ROC is still controversial; in this setting, our policy is directed to perform surgery when one or at most two relapses are detected. This strategy, even if not standardized, is supported by the evidence of improved survival outcomes reported in the literature [3,8]. Nevertheless, we are waiting for the results of the Desktop III trial, which will definitively clarify the effectiveness of such an approach.

This case shows that a 3cm right subdiaphragmatic lesion was initially characterized at CT and PET/CT imaging as an isolated ovarian cancer relapse. Unexpectedly, the lesion was a nonabsorbable propylene suture granuloma at final histologic report and unknown peritoneal carcinosis was found at laparotomy as well. The involvement of the diaphragmatic region, especially the right hemidiaphragm [9], occurs in approximately more than half of the patients with advanced ovarian cancer. Therefore anatomical, surgical and radiological knowledge of this area should be improved when treating ovarian cancer disease. In recent years, many efforts have been made by the gynecologic oncology community to underline the importance of radical diaphragmatic surgery after liver mobilization in order to completely remove the neoplastic implants and improve survival; however, this kind of surgery is burdened by higher perioperative complications [10,11].

The occurrence of granuloma after surgery with nonabsorbable surgical suture has been rarely described, and it usually occurs with the use of silk suture (Table 1) [5-7]; furthermore it has never been associated with ovarian cancer surgery and with the application of propylene suture (Table 1).

**Table 1:** Cases of granuloma after surgery with nonabsorbable surgical suture reported in the literature

**Reference**	**Primary diagnosis**	**Site of FDG accumulation**	**Diagnostic device**	**SUV max**	**Histology**	**Type of material of suture**
Lim *et al.*[5]	Distal sigmoid colon cancer	Mesentery-mesenteric vein	PET	3.9	Extensive fibrosis with focal giant cell reaction	Silk
	Colorectal cancer	Peritoneum within the small bowel mesentery	PET	3.4	Reactive lymphoid hyperplasia, fibrosis	Silk
Yüksel *et al.*[6]	Lung cancer	Right upper lobe (lung)	PET	3.5	Strands of nonabsorbable heavy suture material	Silk
	Lung cancer	Right lower lobe (lung)	PET	3.5	Inflammatory nodule at the site of prior suturing	Silk
Kikuchi *et al.*[7]	Hypopharyngeal squamous cell carcinoma	Prevertebral region	PET	6.0	Epithelioid cells around the silk suture	Silk
	Oropharyngeal squamous cell carcinoma	Left axilla	PET	4.3	Giant cells around the silk	Silk

*Abbreviations*: *FDG* fluorodeoxyglucose, *PET* positron emission tomography, *SUV* standardized uptake value.

In this case the patient developed a late onset granuloma from nonabsorbable propylene suture, 11 months after primary surgery, without any concomitant symptoms and, of note, with previous negative radiological examinations. A suture granuloma is the aseptic fibrinous response to the inflammatory reaction induced by suture antigenicity and/or by the exacerbation of suture bacterial infection, occurring during primary surgery [6,12]. On pathological examination it is characterized by an accumulation of epithelioid histiocytes and multinucleated giant cells, which typically surround the foreign bodies. In this case, we can suppose that her clinical and immunologic deteriorations, probably related to concomitant disease recurrence, led to the granuloma formation.

The false-positive result on PET/CT scan in our patient is probably the consequence of this localized inflammation process, that might impair the accuracy of the method [6], mimicking malignant tissue glucose uptake in a PET scan.

The second disappointing radiological finding in this case is the absence of peritoneal carcinosis, by both CT and PET/CT scans. An early and accurate diagnosis appears to be a fundamental part of the treatment, especially if SCR is taken into consideration. The best approach for preoperative radiological assessment of ROC is still under investigation, but combined PET/CT has recently gained increasing attention [13,14], with encouraging rates of sensitivity and specificity [15].

PET/CT specificity has been shown to be less than 80% and 70% when detecting abdominal and pelvic lesions respectively. Furthermore, it has also been suggested that PET/CT is unable to depict small-volume disease (lesions 5 to 7mm in size) and miliary or diffuse peritoneal involvement [13]. This mismatch needs to be considered when approaching SCR because according to Chi *et al.*[3], this surgical procedure should not be recommended in the presence of peritoneal carcinomatosis in platinum-resistant or partially-sensitive ovarian cancer. Thus, one can speculate that in our patient, a higher accuracy of radiological assessment would probably have spared an unnecessary surgical procedure.

## Conclusions

This case suggests that the occurrence of granuloma from a foreign body can induce false-positive imaging of recurrence. Hence, clinical details on previous surgical procedures are necessary for adequate interpretation. Although PET/CT is a useful innovative diagnostic modality in finding neoplastic lesions such as recurrence of ovarian cancer, it is still burdened by false positives and low sensitivity, as demonstrated in the present case. It could fail to identify early small cancer lesions so that CA-125 remains the most accurate and sensible tool to gain an early prediction of recurrence of disease.

Any imaging modality only increases the detection of recurrence but cannot be considered definitive. It is recommended to double check such cases to confirm or reject the diagnosis.

## Consent

Written informed consent was obtained from the patient for publication of this case report and accompanying images. A copy of the written consent is available for review by the Editor-in-Chief of this journal.

## Abbreviations

CA: Carbohydrate antigen; CT: Computed tomography; F-FDG: ^18^F-fluorodeoxyglucose; PET: Positron emission tomography; ROC: Recurrent ovarian cancer; SCR: Secondary cytoreductive surgery; SUV: Standardized uptake value.

## Competing interests

The authors have stated explicitly that there are no competing interests in connection with this article.

## Authors’ contributions

LI and CM analyzed and interpreted the patient data regarding the cancer disease and the surgery. LI and CM were major contributors in writing the manuscript. LS participated in the design of the study and helped to draft the manuscript. RI participated in the design of the study and helped to draft the manuscript. CB participated in the design of the study and helped to draft the manuscript. LV, LDF and EB participated in the design of the study and helped to draft the manuscript. AM participated in the design of the study and helped to draft the manuscript. IP and PP conceived of the study, and participated in its coordination. PBP conceived of the study, and participated in its coordination. All authors read and approved the final manuscript

## References

[B1] Güth U Huang DJ Bauer G Stieger M Wight E Singer G Metastatic patterns at autopsy in patients with ovarian carcinoma Cancer 2007 110 1272 1280 10.1002/cncr.22919 17634950

[B2] Park CM Kim SH Kim SH Moon MH Kim KW Choi HJ Recurrent ovarian malignancy: patterns and spectrum of imaging findings Abdom Imaging 2003 28 404 415 10.1007/s00261-002-0046-y 12719914

[B3] Chi DS McCaughty K Diaz JP Huh J Schwabenbauer S Hummer AJ Venkatraman ES Aghajanian C Sonoda Y Abu-Rustum NR Barakat RR Guidelines and selection criteria for secondary cytoreductive surgery in patients with recurrent, platinum-sensitive epithelial ovarian carcinoma Cancer 2006 106 1933 1939 10.1002/cncr.21845 16572412

[B4] Pisano C Bruni GS Facchini G Marchetti C Pignata S Treatment of recurrent epithelial ovarian cancer Ther Clin Risk Manag 2009 5 421 426 10.2147/tcrm.s4317 PMC2695243 19753136

[B5] Lim JW Tang CL Keng GH False positive F-18 fluorodeoxyglucose combined PET/CT scans from suture granuloma and chronic inflammation: report of two cases and review of literature Ann Acad Med Singapore 2005 34 457 460 16123823

[B6] Yüksel M Akgül AG Evman S Batirel HF Suture and stapler granulomas: a word of caution Eur J Cardiothorac Surg 2007 31 563 565 10.1016/j.ejcts.2006.11.056 17223570

[B7] Kikuchi M Nakamoto Y Shinohara S Fujiwara K Tona Y Yamazaki H Kanazawa Y Kurihara R Imai Y Naito Y Suture granuloma showing false-positive finding on PET/CT after head and neck cancer surgery Auris Nasus Larynx 2012 39 94 97 10.1016/j.anl.2011.04.012 21620597

[B8] Benedetti Panici P De Vivo A Bellati F Manci N Perniola G Basile S Muzii L Angioli R Secondary cytoreductive surgery in patients with platinum-sensitive recurrent ovarian cancer Ann Surg Oncol 2007 14 1136 1142 10.1245/s10434-006-9273-8 17195909

[B9] De Iaco P Musto A Orazi L Zamagni C Rosati M Allegri V Cacciari N Al-Nahhas A Rubello D Venturoli S Fanti S FDG-PET/CT in advanced ovarian cancer staging: value and pitfalls in detecting lesions in different abdominal and pelvic quadrants compared with laparoscopy Eur J Radiol 2011 80 98 103 10.1016/j.ejrad.2010.07.013 20688446

[B10] Aletti GD Dowdy SC Podratz KC Cliby WA Surgical treatment of diaphragm disease correlates with improved survival in optimally debulked advanced stage ovarian cancer Gynecol Oncol 2006 100 283 287 10.1016/j.ygyno.2005.08.027 16182350

[B11] Chi DS Zivanovic O Levinson KL Kolev V Huh J Dottino J Gardner GJ Leitao MM Jr Levine DA Sonoda Y Abu-Rustum NR Brown CL Barakat RR The incidence of major complications after the performance of extensive upper abdominal surgical procedures during primary cytoreduction of advanced ovarian, tubal, and peritoneal carcinomas Gynecol Oncol 2010 119 38 42 10.1016/j.ygyno.2010.05.031 20609464

[B12] Kim HS Chung TS Suh SH Kim SY MR imaging findings of paravertebral gossypiboma AJNR Am J Neuroradiol 2007 28 709 713 PMC7977336 17416826

[B13] Prakash P Cronin CG Blake MA Role of PET/CT in ovarian cancer AJR Am J Roentgenol 2010 194 464 470 10.2214/AJR.09.3843 20489063

[B14] Son H Khan SM Rahaman J Cameron KL Prasad-Hayes M Chuang L Machac J Heiba S Kostakoglu L Role of FDG PET/CT in staging of recurrent ovarian cancer Radiographics 2011 31 569 583 10.1148/rg.312105713 21415197

[B15] Chung HH Kang WJ Kim JW Park NH Song YS Chung JK Kang SB Lee HP Role of [^18^F] FDG PET/CT in the assessment of suspected recurrent ovarian cancer: correlation with clinical or histological findings Eur J Nucl Med Mol Imaging 2007 34 480 486 10.1007/s00259-006-0260-x 17089122

